# Integrating e-learning into the educational framework for undergraduate medical training: a Nigerian survey on trainers´ perception and readiness

**DOI:** 10.11604/pamj.2024.49.50.42398

**Published:** 2024-10-22

**Authors:** Adesola Olubunmi Adekoya, Oluwaseyitan Andrew Adesegun, Abiola Omobonike Adekoya, Olufunmilola Olubisi Abolurin, Osaze Ehioghae, Akolade Olukorede Idowu, Kolawole John Sodeinde, Funmilola Tolulope Taiwo, Oyinkansola Oluwajomiloju Babayode, Ibukunolu Olufemi Ogundele, Collins Chijioke Adumah, Tinuade Adetutu Ogunlesi

**Affiliations:** 1 Department of Paediatrics, Babcock University, Ilishan-Remo, Ogun State, Nigeria,; 2 Department of Community Medicine, Babcock University Teaching Hospital, Ilishan-Remo, Ogun State, Nigeria,; 3 Department of Radiology, Olabisi Onabanjo University, Ago-Iwoye, Ogun State, Nigeria,; 4 Department of Surgery, Babcock University Teaching Hospital, Ilishan-Remo, Ogun State, Nigeria,; 5 Department of Internal Medicine, Babcock University, Ilishan-Remo, Ogun State, Nigeria,; 6 Department of Internal Medicine, University College Hospital, Ibadan, Oyo State, Nigeria,; 7 Department of Family Medicine, St. Mary´s General Hospital, Eleta, Ibadan, Oyo State, Nigeria,; 8 Department of Surgery, Olabisi Onabanjo University Teaching Hospital, Sagamu, Ogun State, Nigeria,; 9 Department of Surgery, Obafemi Awolowo University, Ile-Ife, Osun State, Nigeria,; 10 Department of Paediatrics, Olabisi Onabanjo University, Ago-Iwoye, Ogun State, Nigeria

**Keywords:** E-learning, perception, readiness, trainers, undergraduate medical training

## Abstract

**Introduction:**

given the significant disruption in educational activities during the COVID-19 pandemic and the uncertainties about the post-pandemic future, coupled with increasing demand for the healthcare workforce, e-learning may bridge the gap in training medical students. It was imperative to survey the perception and readiness of the trainers on the use of e-learning for undergraduate medical training in Nigeria.

**Methods:**

this cross-sectional study was conducted among teachers of medical students in Nigeria. Using Google Forms, information on socio-demographic details, perception of online medical education, and individual and institutional preparedness to adopt e-learning were obtained. Data analysis was done using SPSS (version 21.0).

**Results:**

there were 300 respondents from 25 to 72 years (mean of 47.1 ± 7.8 years). Most were willing to give core lectures and seminars by e-learning, but only about half (159; 53.0%) had ever delivered any lecture online. Many were against conducting laboratory demonstrations (51%), clinical demonstrations (51.7%), and bedside teachings (54.7%) by e-learning. Few (22.0%) were familiar with assignment management packages. Lack of internet connectivity (69.7%) and poor power supply (69.0%) were the most common barriers to implementing e-learning. There was a significant difference (p < 0.001 for all) in institutional internet provision and accessibility, staff training, and using e-learning for lectures between private and public institutions.

**Conclusion:**

utilization of e-learning for medical education is low in Nigeria, with private institutions significantly outperforming the public sector. Many trainers prefer that the practical and clinical aspects should not be integrated into e-learning. Government, institutions and trainers need to do more to improve the acceptance and utilization of e-learning.

## Introduction

Global health has recently seen a paradigm shift towards strengthening health systems, with particular attention paid to the healthcare workforce due to the pivotal role that the workers play in ensuring the optimal health of the people [1]. Nigeria, the most populous African nation [2], has been identified as being in a healthcare workforce crisis. In 2021, Nigeria was reported to have an abysmal ratio of about four doctors per 10,000 population required to deliver essential health services effectively [3]. Some reasons for this deficit include the underproduction of doctors and other cadres of health professionals partly due to frequent industrial actions by public universities and teaching hospital unions, accreditation-related delays in the medical universities, and brain drain. In addition, the rapidly increasing population with a slower production of health professionals meant there would be poor coverage of human resources for health in the long run.

Unfortunately, the COVID-19 pandemic caused significant disruption in educational and other activities worldwide, and there are still uncertainties about the post-pandemic future. Thus, Information and Communication Technologies (ICT) have been strategically positioned as the arbiters of change in all sectors of human activity, including the educational sector. E-learning as defined by the Commission of the European Communities is the “use of new multimedia technologies and the internet to improve the quality of learning by facilitating access to resources and services as well as remote exchanges and collaboration” [4]. While e-learning has proved valuable in various climes, it is yet to be entirely accepted in much of the developing world, where the burden of healthcare workforce inadequacies is highest [5,6]. This may be due to infrastructural deficit, and low level of expertise among other factors. It is generally agreed that only some courses or disciplines can be effectively taught remotely, especially professional disciplines that require hands-on learning experiences, such as medicine, nursing, and other medical disciplines [7].

Several researchers have suggested "blended learning" as a helpful approach in integrating e-learning into medical education in lower-middle-income countries, using e-learning platforms and technologies to cover theoretic training and optimizing face-to-face sessions to cover practical skills, where necessary [8,9]. Kim *et al*. suggested some innovative ways e-learning can prove helpful in medical training including simulation technology, synchronous learning delivery, and web-based/video conferencing of standardized patients [10]. In 2020, the first virtual ward round was reportedly held in the United Kingdom, with the medical students expressing a satisfactory clinical experience [11].

The perceptions and attitudes of the trainers can affect their utilization of these technologies to improve the learning experience for students by transforming some components of the traditional delivery methods into online learning or developing entirely new teaching methods to be administered online. It was, therefore, imperative to survey the trainers on utilizing e-learning to train medical students. The findings may serve as a reference point for developing policies and action plans to ensure the seamless running of medical education in Nigeria. This is the first study to assess the perception and readiness of trainers of medical students across all the geopolitical regions of Nigeria, in both private and public sectors on integrating e-learning into the educational framework.

## Methods

**Study design:** it was a cross-sectional descriptive study. It was carried out over two years, between August 2020 and July 2022.

**Setting:** the study was carried out in private and public universities in all the geo-political zones of Nigeria. According to the Medical and Dental Council of Nigeria, the professional health regulatory agency for the professions of medicine, dentistry, and alternative medicine in Nigeria, there were 37 fully accredited medical schools in Nigeria, most of which are in the South [12,13]. Undergraduate medical education is divided into three - the pre-clinical years, where students undergo training in the basic medical sciences (anatomy, physiology, and biochemistry); the basic clinical sciences (pathology and pharmacology), and the clinical sciences (internal medicine, surgery, pediatrics, obstetrics and gynaecology, community medicine/public health) with specialized postings such as psychiatry, radiology, anesthesia, family medicine, among others.

**Participants:** consenting trainers of medical students from the Basic Medical Sciences, Basic Clinical Sciences, and Clinical Sciences Department were included in the study. Trainers without university employment were excluded from the study.

**Variables:** the socio-demographic data, perceptions, willingness, and individual as well as institutional preparedness to integrate e-learning into undergraduate medical training were obtained from the participants. Likert scale was used to assess the lecturers´ perception of the various aspects of medical teaching that can be incorporated into e-learning. Preparedness was assessed based on the availability of personal and institutional internet facilities, the presence of an e-learning department, and training on e-learning.

**Data sources/measurement:** a structured questionnaire developed by the researchers was pretested online among 20 lecturers of medical students from different academic cadres. Google Forms, an online survey application, was utilized to obtain the relevant information on the variables. The link to the questionnaire was shared on the various professional WhatsApp platforms, Telegram, and via electronic mail to individuals through the research collaborators and their colleagues in different parts of the country.

**Study size:** the minimum sample size was determined using the Kish formula [14]:


$$n=\frac{Z^{2}pq}{d^{2}}$$

Where, n= sample size, p= prevalence, q= 1-p, d= absolute precision, assuming a confidence interval of 95%, 5% absolute precision, and prevalence of 16%, being the proportion of lecturers that utilized virtual classrooms in a previous Nigerian study [15]. This gave a minimum sample size of 207. Correcting for 20% of non-response or incorrectly filled responses increased the minimum sample size to 259 and rounded to 300.

**Statistical methods:** data was collated using Google Spreadsheet and analyzed using the Statistical Package for the Social Sciences Version 21.0 (IBM SPSS Statistics, IBM Corporation, Armonk, NY, USA). The continuous variables were summarised as means and standard deviations while categorical variables were summarised as frequencies and percentages. The Chi-squared test was used for the statistical test of significance between categorical variables, and p < 0.05 was considered statistically significant.

**Ethical considerations:** the aim of the study was explained to all the participants in the introductory section of the questionnaire, and consent was obtained before the participants filled out the questionnaire. There was no form of coercion, and no identifying information was collected. Ethical approval for the study was obtained from the Babcock University Health Research Ethics Committee (BUHREC314/20).

## Results

**Participants:** a total of 300 respondents were recruited for the study.

**Descriptive data:** the ages ranged from 25 to 72 years, with a mean of 47.1 ± 7.8 years. Two (0.7%) were aged less than 30 years, while three (1.0%) were 70 years and above. Most (77.7%) of them worked in public universities (77.7%) as shown in Table 1. Nearly three-fifths (173; 57.7%) of the respondents had previously occupied leadership positions in their universities and hospitals. The common leadership positions occupied were head of department (118), dean/sub-dean (19), director of clinical services (13), and provost/deputy provost (9), among others.

**Table 1:** the respondents' work settings, faculties, and academic ranks

Respondents' characteristics	Number (%)
**Zonal location of medical school**	
South West	210 (70.0)
South East	12 (4.0)
South South	22 (7.3)
North Central	22 (7.3)
North West	20 (6.7)
North East	14 (4.7)
**Work sector**	
Public	233 (77.7)
Private	67 (22.3)
Faculty	
Basic Medical Sciences	23 (7.7)
Basic Clinical Sciences	31 (10.3)
Clinical Sciences	246 (82.0)
**Academic rank**	
Lecturer II	25 (8.3)
Lecturer I	132 (44.0)
Senior lecturer	69 (23.0)
Associate professor	36 (12.0)
Professor	38 (12.7)

**Outcome data/main results:** the majority (295; 98.3%) had ever received online lectures/teaching, including seminars, webinars, or distance learning courses, whereas only about half (159; 53.0%) had given online lectures to medical students. Most (266, 88.7%) considered e-learning advantageous for medical training in Nigeria. The respondents' perceptions on integrating e-learning into the different aspects of medical teaching are shown in Table 2. The majority agreed with giving core lectures (260, 86.7%) and seminars (277, 92.3%) via e-learning, but most were against conducting lab demonstrations, clinical demonstrations, and bedside teachings by e-learning. Most (295; 98.3%) respondents were willing, to some extent, to integrate e-learning into the training of their undergraduate medical students; however, the degree of willingness varied, with 56 (18.7%) being somewhat reluctant, while 239 (79.7%) were very willing.

**Table 2:** the respondents' perception of incorporating e-learning into different aspects of medical teaching

Aspect of medical teaching	Strongly agree n (%)	Agree n (%)	Neutral n (%)	Disagree n (%)	Strongly disagree n (%)
Core lectures	171 (57.0)	89 (29.7)	18 (6.0)	10 (3.3)	12 (4.0)
Seminars	172 (57.3)	105 (35.0)	12 (4.0)	5 (1.7)	6 (2.0)
Lab demonstrations	31 (10.3)	57 (19.0)	59 (19.7)	62 (20.7)	91(30.3)
Clinical demonstrations	21 (7.0)	72 (24.0)	52 (17.3)	51 (17.0)	104 (34.7)
Bedside teachings	16 (5.3)	41 (13.7)	49 (16.3)	69 (23.0)	125 (41.7)

n: number

Almost all (297; 99.0%) of the respondents had personal webcam-enabled laptops that could be used for e-learning purposes, but only 51 (17.0%) of them had individual multimedia projectors; however, the majority (91.9%) of those who did not have individual projectors could readily get access to it from their department or faculty when needed. In assessing individual preparedness for e-learning, it was found that the majority (>90%) of the lecturers were confident with the use of word processing packages (e.g. Microsoft Word), presentation programs for preparing lectures (e.g. Microsoft PowerPoint), and the use of the internet. However, their confidence levels with video conferencing applications (e.g. Zoom, Skype) were lower (82% were confident). A low familiarity with assignment management packages (e.g. Turnitin) was found, with only 22% of respondents needing more confidence in its use. In comparison, 6.3% could not use it, and 9.0% had not heard about it.

Of the available learning management system software, respondents were most aware of Google Classroom (205; 68.3%), followed by Moodle (81; 27.0%) and Edmodo (77; 25.7%), whereas levels of awareness of Schoology (15; 5.0%) and ClassDojo (13 (4.3%) were low. Similarly, the use of learning management system software was highest with Google Classroom (117; 39.0%), followed by Edmodo (34; 11.3%) and Moodle (33; 11.0%).

Concerning the availability of institutional internet connection (not personally sourced), internet connectivity was reported to be provided in the faculty office by 195 (65.0%) of the respondents, in the departmental office by 179 (59.7%), in personal offices by 146 (48.7%), and in students' lecture/seminar rooms by 118 (39.3%). Two hundred and six (68.7%) respondents reported that their institutions encourage using e-learning platforms to engage with medical students. In comparison, 201 (67.0%) said their institutions had an e-learning department responsible for creating and managing e-learning platforms. However, a functioning e-learning platform anchored by such units was reported by 163 (54.3%) of the respondents.

Less than half (137; 45.7%) of the respondents had received training from their institution on utilizing e-learning. On the other hand, about half (151, 50.3%) of the respondents´ institutions used e-learning for teaching medical students during the COVID-19 lockdown. Less than a quarter (74; 24.6%) of the lecturers perceived accessibility to an uninterrupted internet connection for e-teaching as being very easy, while 58 (19.3%) admitted great difficulty in achieving such.

Table 3 shows the relationship between the willingness of respondents to integrate e-learning into the training of their undergraduate medical students and their socio-demographic characteristics as well as work settings. There was no significant relationship between willingness to employ e-learning and the age group, sex, geo-political zone, work sector, or faculty of the respondents. A comparison of the preparedness and utilization of e-learning between public and private medical schools is depicted in Table 4. There was a significant difference in institutional internet provision and ease of accessibility, having an e-learning department, training of staff on the utilization of e-learning, and using e-learning for lectures during the COVID-19 lockdown among public and private institutions.

**Table 3:** relationship between the willingness of respondents to integrate e-learning into medical training and their socio-demographic characteristics/work settings

	Willingness to integrate e-learning into medical training		
	Not willing at all n (%)	Somewhat willing n (%)	Very willing n (%)
**Age-group**			
<40 years	0 (0.0)	11 (25.6)	32 (74.4)
40-59 years	5 (2.1)	42 (18.0)	186 (79.8)
≥60 years	0 (0.0)	3 (12.5)	21 (87.5)
Statistical comparison	χ2 = 4.45*, p = 0.348		
**Sex**			
Male	3 (1.4)	37 (17.7)	169 (80.9)
Female	2 (2.2)	19 (20.9)	70 (76.9)
Statistical comparison	χ2 = 0.67*, p = 0.715		
**Geo-political zone**			
South West	3 (1.4)	38 (18.1)	169 (80.5)
South East	0 (0.0)	1 (8.3)	11 (91.7)
South South	1 (4.5)	5 (22.7)	16 (72.7)
North Central	0 (0.0)	3 (13.6)	19 (86.4)
North West	1 (5.0)	7 (35.0)	12 (60.0)
North East	0 (0.0)	2 (14.3)	12 (85.7)
Statistical comparison	χ2 = 8.73*, p = 0.558		
**Work sector**			
Public	4 (1.7)	43 (18.5)	186 (79.8)
Private	1 (1.5)	13 (19.4)	53 (79.1)
Statistical comparison	χ2 = 0.04*, p = 0.978		
**Faculty**			
Basic Medical Sciences	0 (0.0)	7 (30.4)	16 (69.6)
Basic Clinical Sciences	1 (3.2)	3 (9.7)	27 (87.1)
Clinical Sciences	4 (1.6)	46 (18.7)	196 (79.7)
Statistical comparison	χ2 = 4.70*, p = 0.319		
**Academic rank**			
Lecturer II	1 (4.0)	5 (20.0)	19 (76.0)
Lecturer I	2 (1.5)	25 (18.9)	105 (79.5)
Senior lecturer	1 (1.4)	11 (15.9)	57 (82.6)
Associate/full professor	1 (1.4)	15 (20.3)	58 (78.4)
Statistical comparison	χ2 = 1.22*, p = 0.976		
**Leadership role**			
No	2 (1.6)	21 (16.5)	104 (81.9)
Yes	3 (1.7)	35 (20.2)	135 (78.0)
Statistical comparison	χ2 = 0.69*, p = 0.708		

**Table 4:** comparative analyses of the preparedness and utilization of e-learning between public and private medical schools

	Work sector	
	Public n (%)	Private n (%)
**Internet availability (provided by the institution) in the personal office**		
Yes	94 (40.3)	52 (77.6)
No	139 (59.7)	15 (22.4)
Statistical comparison	χ2 = 28.93, p <0.001	
**Internet availability (provided by the institution) in students’ lecture/seminar rooms**		
Yes	74 (31.8)	44 (65.7)
No	159 (68.2)	23 (34.3)
Statistical comparison	χ2 = 25.08, p <0.001	
**Does your institution have an e-learning department that creates/manages e-learning platforms?**		
Yes	144 (61.8)	57 (85.1)
No	89 (38.2)	10 (14.9)
Statistical comparison	χ2 = 12.75, p <0.001	
**Have you ever received training from your institution on how to utilize e-learning?**		
Yes	87 (37.3)	50 (74.6)
No	146 (62.7)	17 (25.4)
Statistical comparison	χ2 = 29.16, p <0.001	
**Did your institution utilize e-learning to teach medical students during the COVID-19 lockdown?**		
Yes	87 (37.3)	64 (95.5)
No	146 (62.7)	3 (4.5)
Statistical comparison	χ2 = 70.47, p <0.001	
**How would you rate your ease of accessibility to an uninterrupted internet connection for e-teaching?**		
Very easy	38 (16.3)	36 (53.7)
Not so easy	140 (60.1)	28 (41.8)
Very difficult	55 (23.6)	3 (4.5)
Statistical comparison	χ2 = 42.50, p <0.001	

Lack of internet connectivity (209; 69.7%) and poor power supply (207; 69.0%) were the most common perceived barriers to implementing e-learning, as reported by the study participants. Others were the cost of setting up e-learning facilities (150; 50.0%), lack of specific software for e-learning (149; 49.7%), lack of willingness to adopt new methods of teaching (135; 45.0), and lack of required equipment (e.g. laptops) by students (129; 43.0%).

## Discussion

Before the COVID-19 pandemic, the utilization of e-learning in sub-Saharan African institutions was remarkably low, as traditional face-to-face training was predominant, especially in medical training [15]. To keep up with the ever-rising need for medical doctors in developing nations, novel methods of continued education amidst several barriers have facilitated an increasing acceptance and utilization of e-learning in Nigerian medical institutions. Despite this, the overall level of utilization of e-learning in Nigerian institutions remains low compared to developed nations [15].

**Key results/interpretation:** this study assessed the perception, utilization, and readiness of trainers to integrate e-learning into different aspects of undergraduate medical training in Nigeria. The trainers perceived e-learning as a veritable learning platform. However, at the time of this study, the utilization of e-learning via online lectures was low. Only about half of the respondents had ever delivered any lecture online despite 98.3% having received online educational activities. This is further evidence of the low utilization of e-learning in developing nations.

Due to the peculiarity of bedside teachings, theatre sessions, patient clerking, and case presentations, it may be more challenging to cover these clinical aspects of medical education solely by e-learning. Hence, a blend of conventional face-to-face teaching and e-learning is widely recommended [16-18]. In agreement, our study revealed that most trainers were willing to conduct core lectures and seminars by e-learning. However, most were against conducting lab demonstrations, clinical demonstrations, and bedside teachings by e-learning. The gradual acceptance and utilization of the innovative virtual ward round may be the ultimate game changer in the practical components of undergraduate medical education [11].

A significantly higher level of preparedness and utilization of e-learning was seen in trainers from the private sector, despite a comparable level of willingness to integrate e-learning into medical training in the public sector. A similar observation was made in Uganda where a significant difference was observed among lecturers in private and public universities in readiness to adopt e-learning [19]. This is probably due to the self-financing nature of private institutions which may influence greater operating agility and make decisions faster, as opposed to public institutions in which decision-making and implementation may be much slower [15].

Barriers to the adoption and utilization of e-learning are indeed multifactorial. A major challenge facing the utilization of e-learning by the lecturers is the unwillingness and lack of drive by the trainers to change their teaching methods [20]. This is particularly true in institutions primarily populated by elderly trainers, as younger trainers tend to be more computer-literate. In our study, however, age, work sector, geographical location, faculty, and academic rank had no significant relationship with the willingness to integrate e-learning into medical training.

Other common barriers to using e-learning include inadequate internet facilities and inadequate training of trainers and students [21-23]. Lack of internet connectivity and poor power supply were the most common barriers to implementing e-learning in this study. Other barriers included the cost of setting up e-learning facilities, lack of specific software for e-learning, lack of willingness to adopt new teaching methods, and lack of required equipment. The lack of essential amenities to ensure the seamless running of e-learning perpetually plagues Nigerian higher institutions. In this aspect, the public sector is significantly affected due to poor allocation of funds from the government [24].

Most of the trainers in our study were confident in using word processing packages and the internet. However, they were not confident using video conferencing applications and assignment management packages. Considering learning management systems, Google Classroom was the most utilized platform. This was similar to previous observations [25,26]. These systems and applications are integral to distance learning. Again, amidst these barriers, the private sector had much better bearing. Individual preparedness cannot be overemphasized; hence, proper training and personal development of staff and students are essential in improving the utilization of e-learning.

Due to the barriers, many medical trainers may prefer conventional classroom teaching to e-learning. Since conducting lectures, seminars, and presentations via e-learning appears more practicable, the basic medical and basic clinical years may be more seamless via e-learning. Regular training and re-training sessions need to be organized for trainers to maximize the benefits of e-learning. If well harnessed, digital platforms may become useful for undergraduate medical examinations. This may be a line of research in the future.

**Strength and limitation:** a limitation of our study is the relatively limited cross-sectional design which precludes follow-up of the trainers after significant exposure and training on e-learning. In addition to response bias, the online data collection also meant that the responses may not be evenly distributed across geographical regions because most responses were from the southwest. Further studies to evaluate the utilization of e-learning in medical education post-training will elucidate additional factors that may militate against the effective utilization of e-learning in undergraduate medical training in Nigeria.

## Conclusion

A low utilization of e-learning was observed in this study, with the private institutions significantly outperforming the public institutions. Barriers to the implementation of e-learning included poor internet and power supply, high cost of setting up e-learning facilities, lack of specific software for e-learning, and lack of training. Intentional efforts by the government and institutions are important to provide adequate equipment and training towards delivering a good mix of conventional face-to-face and e-learning-facilitated training to improve undergraduate medical training.

### What is known about this topic

- E-learning is inevitable in medical education;
- E-learning is more acceptable in developed countries than in developing ones;
- The blended learning approach is the widely practiced method of medical education.

### What this study adds

- Many trainers are willing to integrate e-learning into the theoretical, but not the practical and clinical aspects of medical education;
- Our data showed that the utilization of e-learning is low, and most trainers were unfamiliar with assignment management packages;
- In terms of institutional commitment, preparedness, and utilization of e-learning, the private medical training institutions in Nigeria are faring much better than the public ones.
